# False discovery rate control in cancer biomarker selection

**DOI:** 10.1016/j.gendis.2022.12.010

**Published:** 2023-01-20

**Authors:** Zhaoming Li

**Affiliations:** Department of Oncology, The First Affiliated Hospital of Zhengzhou University, Zhengzhou, Henan 450052, China

We read with great interest the manuscript “LPCAT1 functions as a novel prognostic molecular marker in hepatocellular carcinoma” by Zhang et al in a recent issue of *Genes & Diseases*.^1^ The authors conducted bioinformatics analyses using high throughput RNA sequencing data from TCGA to demonstrate that LPCAT1 is a novel and effective prognostic marker for hepatocellular carcinoma. We appreciate the contributions of the authors on the subject, nonetheless, we have some concerns that should be clarified in the following issues.

The standard *P*-value was invented for testing individual hypotheses. There is an obvious problem when analyzing gene expression data collected via sequencing of multiple genomes, as this usually involves testing from several thousands to tens of thousands of hypotheses simultaneously. In genome sequencing studies most researchers are keenly aware of the potentially high rate of false positives and the need to control it. One key statistical shift is the move away from the well-known *P*-value to false discovery rate (FDR).^2^^,^^3^ The FDR of a test is defined as the expected proportion of false positives among the declared significant results.^3^, ^4^, ^5^ Because of this directly useful interpretation, FDR is a more convenient scale to work on instead of the *P*-value scale. However, in Zhang's report, the multiple test correction was not applied to the *P* values shown in Figure 2A and 7 and stated in the text. This seems to be required since the authors tested the association between each gene and outcome individually.

The correlation between gene expression and overall survival in the same hepatocellular carcinoma RNA sequencing data from TCGA were re-analyzed by the Genomics Analysis and Visualization Platform (http://r2.amc.nl) and the results were corrected for multiple gene testing by FDR. The potential LPCAT1-related tumor genes reported by the author and their adjusted *P*-value were provided in Table 1. It showed that the expression of several genes (CCNB2, CENPF, and UBE2C) had no associations with overall survival, which is different from Figure 7 in the report.

**Table 1:** The association of LPCAT1-related tumor genes with overall survival. Results were corrected for multiple gene testing by false discovery rate.

No.	Gene	Probeset	Adjusted *P*-value
1	*CDC20*	CDC20_991	0.001464779
2	*CDCA8*	CDCA8_55143	0.005409055
3	*LPCAT1*	LPCAT1_79888	0.00569335
4	*TPX2*	TPX2_22974	0.00691434
5	*DLGAP5*	DLGAP5_9787	0.008015183
6	*CCNB1*	CCNB1_891	0.008149167
7	*MAD2L1*	MAD2L1_4085	0.009098616
8	*KIF4A*	KIF4A_24137	0.00910015
9	*NUF2*	NUF2_83540	0.009505066
10	*CENPA*	CENPA_1058	0.011305291
11	*KIF11*	KIF11_3832	0.011942813
12	*KIF20A*	KIF20A_10112	0.012491643
13	*BIRC5*	BIRC5_332	0.013002953
14	*KIF2C*	KIF2C_11004	0.013481529
15	*CDK1*	CDK1_983	0.015119904
16	*TTK*	TTK_7272	0.018113202
17	*PLK1*	PLK1_5347	0.018869319
18	*BUB1*	BUB1_699	0.023996996
19	*BUB1B*	BUB1B_701	0.025563704
20	*RRM2*	RRM2_6241	0.025577274
21	*NCAPG*	NCAPG_64151	0.029011476
22	*TOP2A*	TOP2A_7153	0.029439732
23	*NDC80*	NDC80_10403	0.029498546
24	*RACGAP1*	RACGAP1_29127	0.030345955
25	*KIF18A*	KIF18A_81930	0.035375811
26	*CEP55*	CEP55_55165	0.036683405
27	*CENPE*	CENPE_1062	0.041494437
28	*AURKB*	AURKB_9212	0.047951168
29	*CCNB2*	CCNB2_9133	not significant
30	*CENPF*	CENPF_1063	not significant
31	*UBE2C*	UBE2C_11065	not significant

## Conflict of interests

The author declares no potential conflict of interests.
